# Modeling COVID-19 spread using multi-agent simulation with small-world network approach

**DOI:** 10.1186/s12889-024-18157-x

**Published:** 2024-03-02

**Authors:** Qin Fan, Qun Li, Youliang Chen, Jianbo Tang

**Affiliations:** 1 School of Civil and Surveying & Mapping Engineering, Jiangxi University of Science and Technology, Ganzhou, 341000 China; 2 Urban Planning Desion Institute of Ganzhou, Ganzhou, 341000 China; 3 Department of Geo-informatics, Central South University, Changsha, 410000 China

**Keywords:** COVID-19, Agent, Small world networks, Social relationship, Spatiotemporal propagation

## Abstract

**Background:**

The rapid global spread of COVID-19 has seriously impacted people’s daily lives and the social economy while also posing a threat to their lives. The analysis of infectious disease transmission is of significant importance for the rational allocation of epidemic prevention and control resources, the management of public health emergencies, and the improvement of future public health systems.

**Methods:**

We propose a spatiotemporal COVID-19 transmission model with a neighborhood as an agent unit and an urban spatial network with long and short edge connections. The spreading model includes a network of defined agent attributes, transformation rules, and social relations and a small world network representing agents’ social relations. Parameters for each stage are fitted by the Runge-Kutta method combined with the SEIR model. Using the NetLogo development platform, accurate dynamic simulations of the spatial and temporal evolution of the early epidemic were achieved.

**Results:**

Experimental results demonstrate that the fitted curves from the four stages agree with actual data, with only a 12.27% difference between the average number of infected agents and the actual number of infected agents after simulating 1 hundred times. Additionally, the model simulates and compares different “city closure” scenarios. The results showed that implementing a ‘lockdown’ 10 days earlier would lead to the peak number of infections occurring 7 days earlier than in the normal scenario, with a reduction of 40.35% in the total number of infections.

**Discussion:**

Our methodology emphasizes the crucial role of timely epidemic interventions in curbing the spread of infectious diseases, notably in the predictive assessment and evaluation of lockdown strategies. Furthermore, this approach adeptly forecasts the influence of varying intervention timings on peak infection rates and total case numbers, accurately reflecting real-world virus transmission patterns. This highlights the importance of proactive measures in diminishing epidemic impacts. It furnishes a robust framework, empowering policymakers to refine epidemic response strategies based on a synthesis of predictive modeling and empirical data.

## Background

Since the outbreak of COVID-19, various prevention and control measures have been implemented in different regions worldwide. However, the persistent spread of COVID-19 continues to represent a significant challenge for many countries. Several studies have established disease models that aim to predict the development of the epidemic. Currently, the more frequently used epidemiological modeling methods include the propagation dynamics model, cellular automata model, and multi-agent model. In the history of epidemiological research, Kermack, et al. [1] proposed the famous SIR compartment model, which laid the foundation for studying epidemic transmission dynamics models. The basic idea is to divide the population into different classes (compartments), which represent the population’s various disease states. Mathematical methods are subsequently employed to establish dynamic equations of these variables and then investigate the dynamic process of disease transmission. Later, many scholars have improved the classical compartment model to better reflect the actual situation of the disease. For example, Jana, et al. [2], Khan, et al. [3], Amaro [4], Zhu, et al. [5], Yang, et al. [6] have improved the basic warehouse model for prediction analysis. However, the infectious disease dynamics model significantly simplifies the simulation process and does not consider the heterogeneity of the population. The cellular automata model was proposed by Von Neumann and Ulam in 1948 and is often employed to simulate the spatiotemporal evolution of complex phenomena. Bin, et al. [7] used the cellular automata model to simulate and analyze the application of influenza A (H1N1). Although the epidemic simulation model based on cellular automata overcomes population heterogeneity, it does not consider human movement and behavior. The agent model can effectively describe individual behavior, making it widely used in simulating the spread of various diseases, such as COVID-19 [8, 9], malaria [10, 11], AIDS [12], SARS [13], Ebola virus [14]. However, the agent model also does not take into account the impact of social relations between agents on the epidemic’s spread according to Wang, et al. [15].

In the transmission of an epidemic, people are a critical source of infection, and their social relationships significantly impact on the epidemic’s spread. Watts, et al. [16] proposed small-world networks to describe human social connections. The emergence of complex network theory offers a fresh perspective for conducting dynamic simulations of epidemics. More and more studies use complex networks to simulate disease transmission and evaluate the effectiveness of prevention and control measures [17–21]. Liu, et al. [19] used contact and contactless networks to simulate the two-stage outbreak of COVID-19 on the Diamond Princess cruise ship. Alrasheed, et al. [18] proposed a network-based epidemic model to simulate the spread of COVID-19 in Saudi Arabia. They used a variety of scenarios to predict the epidemic dynamics of Saudi Arabia in the next 6 months. Peirlinck, et al. [20] combined the network model with the SEIR model to predict the peak of the COVID-19 outbreak in China and the United States. The current epidemic spread based on the network model focuses more on the influence of parameters such as infection rate and primary reproduction number on epidemic spread combined with the compartment model [22, 23]. The results are the trend of the epidemic in time. However, the spread of epidemics is a time and spatial evolution process [24]. Since the outbreak of COVID-19, some scholars have studied the risk of COVID-19 infection [25, 26] as well as the spatial and temporal distribution characteristics [27–30]. Meanwhile, some scholars pay attention to the spatial characteristics of epidemics during the transmission process. However, most spatiotemporal modeling ignores individuals’ social relations [31]. It should be noted that individual behavior and government intervention can significantly impact the spread of epidemics.

Given the aforementioned issues, this study proposes a small-world network and multi-agent collaborative COVID-19 spatiotemporal propagation simulation model to simulate the spread of COVID-19 for urban areas. The neighborhood is regarded as a kind of agent with the exact nature, and the interaction between the communities is realized by establishing a small-world network. By integrating individuals’ social connections into the spatiotemporal modeling of COVID-19 spread, alongside GIS (Geographic Information System), data visualization, and other technologies, this approach fully investigates various information relationships and describes the epidemic’s temporal and spatial propagation. Taking Wuhan urban area as an example, this model simulated the early stages of COVID-19 outbreak. Multiple scenarios were developed to simulate the epidemic’s evolutionary trends, and analyzed and discussed using the simulation results. The data simulations confirmed the excellent applicability of this model.

## Methods and study design

In this study, we use the neighborhoods as the primary unit of investigation. In order to represent the activity relationships between neighborhoods, we designed a spatiotemporal simulation model containing both short and long connections. Short connections were established based on interactions within the same activity, and connections between neighborhoods were facilitated due to the activities of individuals within them. On the other hand, long-lasting connections were established between distant neighborhoods where individuals engage in common activities.

### COVID-19 spatiotemporal propagation simulation model

In the context of the COVID-19 pandemic, it is crucial to determine various epidemiological parameters for the disease accurately. To achieve this, we utilized the Runge-Kutta method and fitted the fmincon function in MATLAB to minimize the sum of squared residuals, resulting in optimal parameter values. These values were then integrated into the SEIR (Susceptible-Exposed-Infectious-Removed) model to predict the number of COVID-19 cases. It is worth noting that the transmission of the virus primarily occurs among acquaintances within individuals’ daily activities, emphasizing the importance of understanding social networks in disease transmission.

#### Agent model

The agent model mainly consists of agents with specific action objectives, which can perceive the environment and decision-making behavior under certain conditions. By defining the attributes and behaviors of agents, some phenomena in the real world can be simulated. Agents may represent a single individual or a homogeneous class of individuals. When constructing infectious disease models, modeling objects are mainly divided into microscopic individuals and single/mixed groups [32]. The microscopic individual considers a single individual as the research object while taking into account differences between individuals. In contrast, the single group regards a class of individuals with the same characteristics and explores the differences between individuals with different characteristics. The composite group represents individuals living in a relatively independent geographical area and the migration of internal individuals links the sub-groups. In order to explore the epidemic situation of COVID-19 in urban areas, this paper adopts the mixed group method, where the population in the neighborhood is regarded as a sub-group, the agent represents all the individuals in a single cell, and the network represents the connections between cells due to the movement of the internal individuals.

In our study, the agent-based model adopts the population partitioning method of the SEIR (Susceptible-Exposed-Infectious-Removed) model to characterize the dynamics of disease transmission at the neighborhood level. Within this model framework, an individual “agent” does not represent a single person but rather a collective of residents within a neighborhood. Specifically, a “Susceptible Agent” (S) denotes a state where there has been no disease transmission within the neighborhood; an “Exposed Agent” (E) signifies that residents within the neighborhood have been exposed to the pathogen, though they have not yet exhibited symptoms but carry the risk of infection; an “Infectious Agent” (I) describes the presence of at least one resident within the neighborhood displaying symptomatic infection; and a “Removed Agent” (R) encompasses all residents who have recovered or passed away due to the disease. Transitions between states, such as from “Exposed” to “Infectious,” indicate the progression of illness in at least one resident within the neighborhood, reflecting the overall health status of the population within that neighborhood. Defining agent attributes, social relations, and state transition rules construct the agent model of COVID-19. The agent attributes can be described as follows:

Definition 1:

Agent attributes. Agent attributes refer to the properties of agents. The agent attributes of this paper include agent identification, agent location, latent days, infection days and agent category, which are expressed as:

1 $$A=\left(O,P,{D}_e,{D}_i,K\right),$$

In Eq.1, *O* represents the number of the agent, *P* denotes the geographical location of the agent in the virtual space, *D*_*e*_ represents the number of days when the latent agent is in the incubation period, *D*_*i*_ represents the number of days when the infected agent is in the infection period, *K* represents the type of agent, that is, an agent at a particular time belongs to the susceptible agent, the latent agent, the infected agent or the evacuee agent. It is crucial to note that each agent can only belong to a particular type of agent at each time.

#### Simulation of COVID-19 spatiotemporal propagation of small-world networks with cooperative multi-agent

The impact of interpersonal relationships on the prevalence of epidemics is significant. Individuals tend to have fixed social networks with stable relationships in everyday life. Viral infections are usually spread among acquaintances in these networks. Understanding the patterns of viral transmission within individual social networks is crucial for controlling the epidemic. The small-world network model is a widely used method for describing social relationships between people. In this paper, we use the small-world network to model these relationships and construct a spatiotemporal simulation model called the COVID-19 Small-World Network Collaborative Multi-Agent Model. This model combines the small-world network approach with multi-agent modeling techniques to simulate the spread of COVID-19.

The small-world network model captures the clustering and separation of nodes in real-world systems. Within social networks, this property means that individuals who do not know each other can be connected by short chains of acquaintances, leading to the small-world phenomenon. Many empirical network diagrams exhibit small-world phenomena, such as social networks, the underlying architecture of the Internet, Wikipedia’s encyclopedia sites, and genetic networks. The clustering coefficient and average path length are key parameters that characterize small-worldness in a network, helping to determine whether it possesses such characteristics. The clustering coefficient measures the proximity of neighbor nodes, while the average path length indicates the typical distance between any two nodes in the network. A small-world network falls between regular and random networks, with a significant clustering coefficient and a small path length. The nodes in the small-world network can represent the agents, and the connection between the nodes can represent the social relationship between the agents.

Our refined model integrates the dynamic characteristics of small-world networks to better understand COVID-19 spread through social connections. It highlights the importance of the network’s evolving nature and the non-uniformity of connections, where some nodes have more significant interactions than others. This complexity, reflecting multiple layers of relationships, is crucial to depict the intricate viral transmission patterns in extensive social networks. The model focuses on key features like the high clustering coefficient and short average path lengths, demonstrating how close-knit groups and short connection chains between individuals can accelerate the spread of the virus. This approach aims to offer a more detailed and realistic simulation of epidemic propagation, underscoring the influence of social network structures on disease dynamics. To understand the role of social connections in spreading the COVID-19 epidemic, the number of agents ‘neighbors and the average degree of the network are defined as:

Definition 2:

**Number of agent neighbors.** The number of edges directly connected to the agent node *i*, that is, the degree (*U*_*i*_) of node *i*, is expressed as:

2 $${U}_i=\sum \limits_{b\in L}{A}_i^b,$$

In Eq. 2, *L* is the set of all sides; ${A}_i^b$ takes a value of 1 or 0, mainly determines whether *b* contains node *i*; if it does, ${A}_i^b$ value takes 1, otherwise take 0. Generally, a more extensive *U*_*i*_ indicates that the node is more important in the network.

Definition 3:

**Network average degree.** The average degree of all agents in the network is the average degree of the network (<*k*>), expressed as:

3 $$<\upkappa \ge \frac{\sum {U}_i}{N},$$

In Eq. 3, *N* denotes the number of nodes in the network, and *U*_*i*_ denotes the number of neighbors of node *i*. Jia, et al. [31] proposed constructing a small-world network by “random edging.” They used this method to establish an agent model of the small-world network to simulate the social relationship between agents.

Definition 4:

Agent social relationship. The connection between the nodes indicates the social connection between the agents. If there is a connected edge between the agents, it indicates that there is a social relationship between the two agents. Otherwise, there is no social relationship, expressed as:

4 $$w={A}_{ij}^l,$$

In Eq. 4, *i* and j represent node *i* and node *j* in the network. The parameter *w* denotes the presence of a connection between these nodes: it is set to 1 if there is an edge, here labeled as *l*, connecting node *i* to node *j*; and it is set to 0 if no such edge exists.

In this study, we constructed a network of 4671 nodes, each corresponding to a neighborhood. The average degree of 6.8 indicates that each neighborhood is directly connected to 6.8 other neighborhoods. The average path length of the network is 6.4 steps, demonstrating that neighborhoods can reach each other through a few intermediary steps, even within a large-scale network. Moreover, the average clustering coefficient of the network is 0.28, which is significantly higher than the expected clustering coefficient of a random network of the same size, indicating a tendency for nodes within the network to form highly clustered groups.

**Fig. 1:**
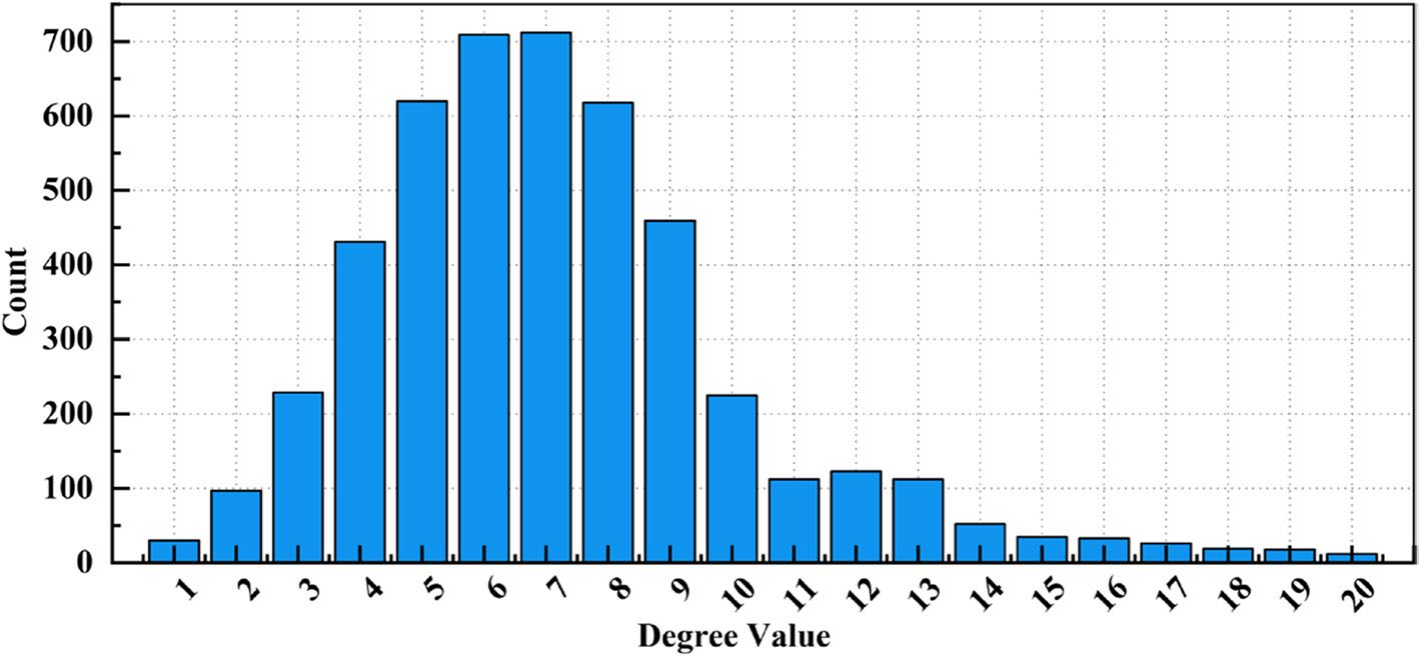
Degree distribution chart

**Fig. 2:**
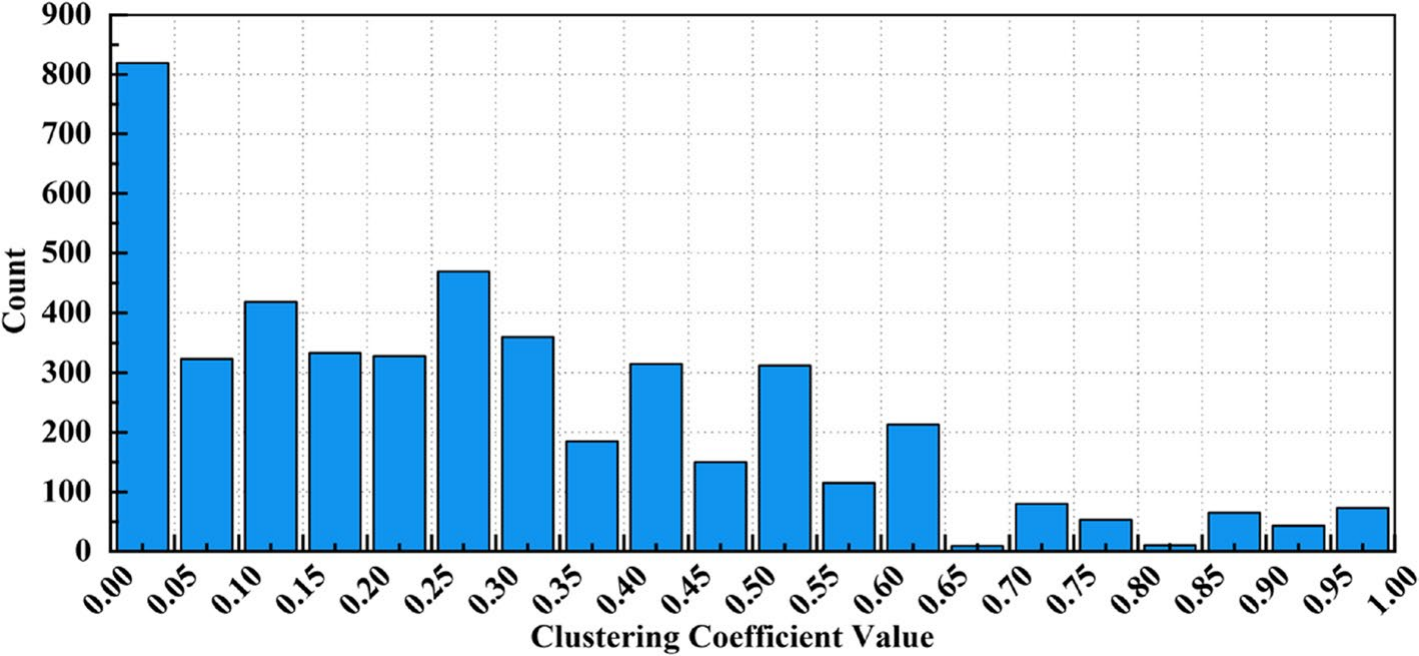
Clustering coefficient distribution chart

Figure 1 displays the degree distribution of the network’s nodes, where most nodes have relatively low degree values while a few have high degrees. This distribution pattern aligns with the typical degree distribution characteristics of small-world networks, where most nodes are interconnected through a few highly connected nodes. Figure 2 shows the distribution of the clustering coefficients of the nodes. The higher peaks of the clustering coefficients suggest strong interconnections between nodes within the network, reflecting the high clustering coefficient characteristic of small-world networks.

Combining these observations, it is evident that the network of the study area exhibits two critical features of small-world networks: one is the short average path length, allowing any two nodes to be reached through a limited number of intermediary nodes; the second is the high clustering coefficient, meaning that nodes tend to form tight-knit groups. These features together support the conclusion that the study area possesses small-world network properties.

**Fig. 3:**
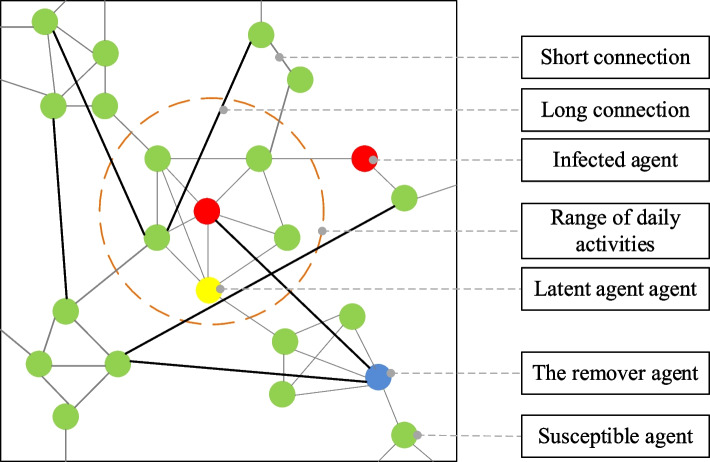
Structure of the COVID-19 propagation model

In an agent model using a small-world network, the social relationship between agents is expressed by constructing short and long connections based on the network’s topology. A short connection is randomly established within a specific distance, representing an activity range of people’s daily lives. The long connection selects an agent with a more significant node degree to connect randomly with other agents, reflecting far commuting or participation in large-scale activities. Far-distance cells are connected when internal individuals participate in the same activity. Figure 3 illustrates the structure of the COVID-19 propagation model, where green circles represent susceptible agents, yellow circles represent latent agents, red circles represent infected agents, and blue circles represent removed agents.

The steps to build the social network of the agent is as follows:

1. According to the range of people’s daily activities, the amicable relationship between the daily activities of the agent is randomly established.
2. Select an agent with a certain degree of high modality and randomly connect with other agents to construct a distant relative relationship for long-distance commuting. Barabási, et al. [33] proposed the “preference dependency” network model, emphasizing that the probability of connecting edges between nodes in real networks often has the characteristics of “heavy-tail distribution,” and subsequent studies have also shown that this network structure has essential applications in epidemic transmission and cluster behavior [34, 35].

#### Propagation mechanism

**Fig. 4:**
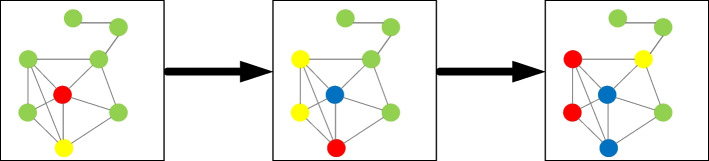
Agent state transition rules

The state of an agent can change at any time. Disease transmission mainly occurs between agents through short and long connections. Infected and latent agents are infectious, and those in contact with them within the infection period have a certain probability of contracting the disease. Susceptible agents can be infected by either infected or latent agents and become latent agents. Latent agents will transition to infected after incubation, and infected agents will become removed agents after the end of the infection period, which prevents them from being reinfected. The state transition rules of the agent are shown in Fig. 4.

**Fig. 5:**
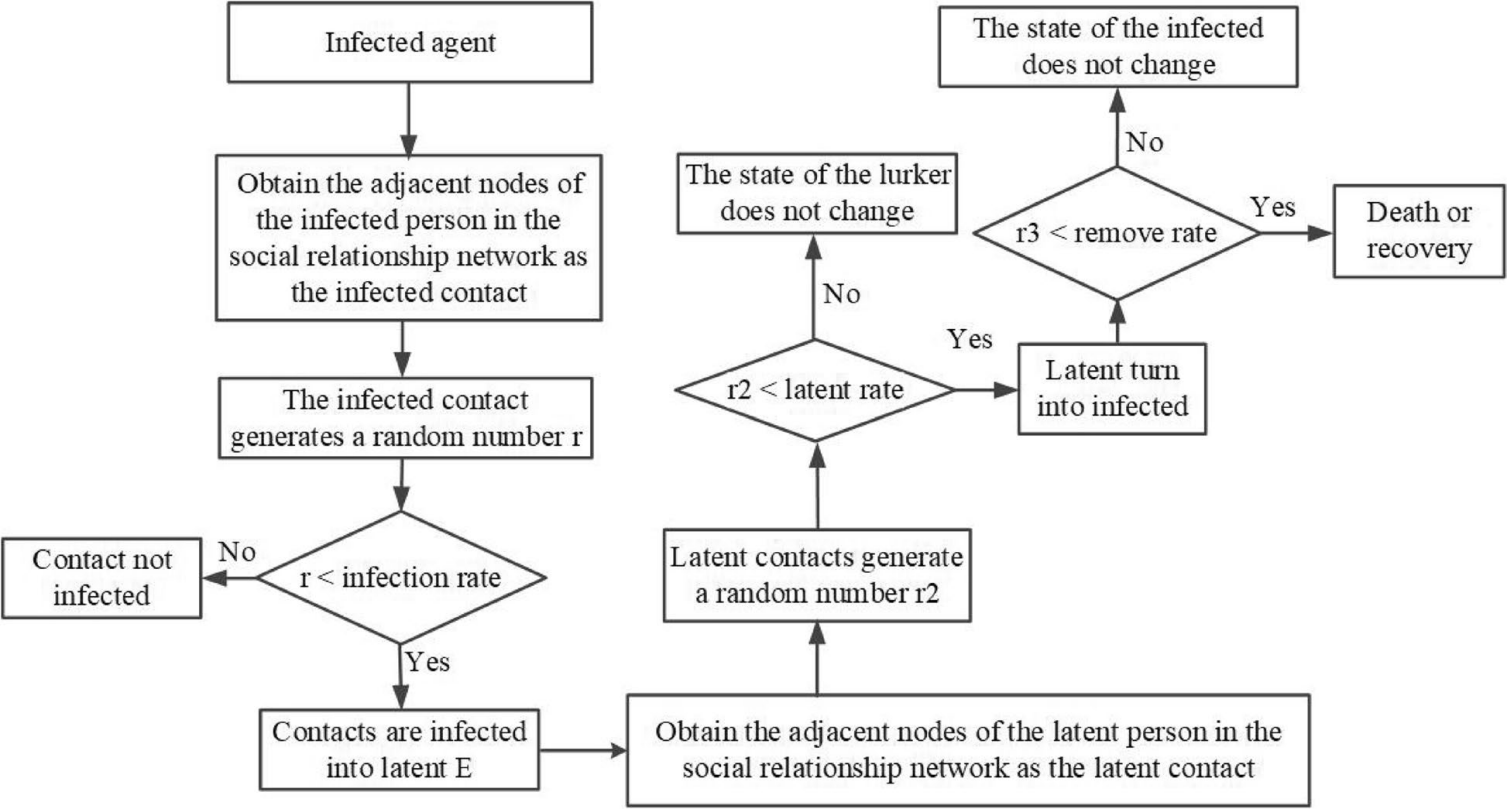
Agent propagation mechanism

The agent propagation mechanism is shown in Fig. 5. Firstly, the adjacent agents of the infected agent in the social relationship network are obtained as the infected contacts. The infected contacts generate a random number *r* to compare with the actual infection rate to determine whether the infected contacts are infected with the latent agent. If the random number *r* exceeds the infection rate, the infected contacts are not infected. Conversely, if the random number *r* is less than or equal to the infection rate, the infected contacts are infected into the latent agent. Similarly, the latent agent obtains the adjacent agent of the latent person in the social relationship network as the latent contact. The latent contact generates a random number *r2* to compare with the latent rate to determine whether the latent contact ends the latent contact period and becomes the infected agent. If the random number *r2* exceeds the latent rate, the latent contact remains in the latent period. Otherwise, the random number *r2* is less than or equal to the latent rate, and the latent contact ends the latent period and becomes the infected agent. During the treatment process, an infected agent generates another random number, denoted as *r3*. This random number is compared to the removal rate to determine whether the infected agent recovers or succumbs to the disease. If the random number *r3* is less than or equal to the removal rate, the infected agent transitions to the removed state, representing recovery or death. However, if the random number *r3* is greater than the removal rate, the state of the infected agent remains unchanged.

#### Model simplification

To simplify the implementation of the COVID-19 space-time propagation model using small-world network collaborative multi-agent, several assumptions are proposed:

1. It is assumed that the infection and the removal probability between cells are the same without considering the impact of factors such as the internal size of the cell.
2. All lurking agents will be transformed into infected agents, and there are no cases of self-healing or death of lurking agents or reinfection of removed agents.
3. Neighborhood social network relationships are mainly concentrated within a specific range of surrounding communities. If a city is under comprehensive control management and all public transport in the urban area is closed, remote communities have no social relationships. When a cell is under closed management, all connections are removed, indicating no social relationship between cells.
4. Epidemic transmission only spreads through cell connection networks. If there is a network connection between the cells, the connected cells are likely to be infected and become epidemic cells if there is an epidemic cell.

#### Model realization

The pseudocode for the COVID-19 spatiotemporal propagation model using small-world network collaborative multi-agent is shown in Table 1.

**Table 1:**
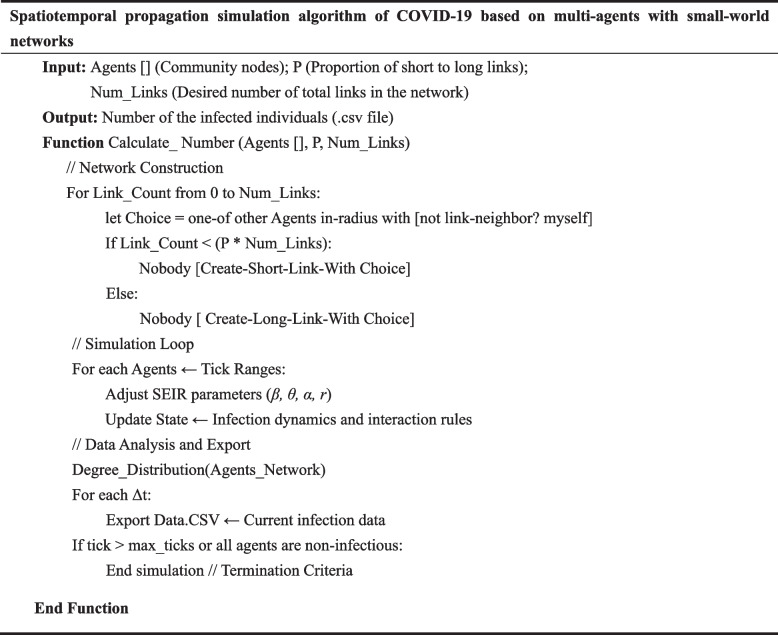
Spatiotemporal propagation simulation algorithm of COVID-19

Step 1: Initialization, mainly including agent initialization and space environment initialization. Agent initialization configures the four types of agents S, E, I, and R, according to the agent properties and methods for the acquired agent information; Space environment initialization is to process boundary data to provide a space environment basis for the activities of agents;

Step 2: Build a virtual space. Load cell vector data and urban vector data in NetLogo to build virtual space;

Step 3: Parameter Estimation. Utilizing real COVID-19 data, the SEIR model parameters were estimated by fitting the model using the Runge-Kutta method to forecast COVID-19 trends. Subsequently, MATLAB’s built-in function fmincon was employed to optimize the parameters, aiming to minimize the sum of squared residuals and thereby obtain fitted values for parameters *β*, *θ*, *α*, and *γ*. Fmincon is a function designed to solve constrained nonlinear minimization problems, which is particularly suitable for our model due to its effective handling of complex constraints. The choice of this function was based on its efficiency and accuracy in dealing with such optimization problems. Specific configurations were made to the parameters of fmincon to ensure optimal fitting of parameters *β*, *θ*, *α*, and *γ*, while adhering to the model constraints. The specific steps are as follows:

1. Considering that the COVID-19 has an incubation period, this paper selects the SEIR model for simulation. The specific formula of the SEIR model is as follows:

5 $${\displaystyle \begin{array}{l}\frac{dS(t)}{dT}=\frac{-\beta I(t)S(t)-\theta E(t)S(t)}{N},\\ {}\frac{dE(t)}{dT}=\frac{\beta I(t)S(t)+\theta E(t)S(t)}{N}-\alpha E(t),\\ {}\frac{dI(t)}{dT}=\alpha E(t)-\gamma I(t),\\ {}\frac{dR(t)}{dT}=\gamma I(t),\end{array}}$$

In Eq. 5, *N* is the total number of people, and *S(t)*, *E(t)*, *I(t)*_**,**_ and *R(t)*, respectively, represent the total number of susceptible, latent, infected, and recovered people at time *t*, and *S(t) + E(t) + I(t) + R(t) = N* is satisfied at any time, which means that the total number of four types of people at time *t* is the total number of people, and remains unchanged; *β* encapsulates the transmission probability of the virus by infected individuals during their interaction with susceptible ones, whereas *θ* corresponds to the likelihood of virus dissemination by exposed individuals in contact with susceptible populations.; *α* indicates the probability that the latent person will be transformed into an infected person, and *γ *is the removal rate.

(2) It should be noted that the model parameters in Eq. (5) are not directly estimated from data. In this study, we employed the fourth-order Runge-Kutta method for numerical solving, which is suitable for systems of ordinary differential equations and has been proven to provide excellent solving performance [36]. With known derivatives of the equations and initial values, the fourth-order Runge-Kutta method effectively simplifies the process of solving differential equations, particularly in computer simulation applications. Therefore, we used this method to numerically solve the SEIR model in Eq. (5) to obtain the model parameters. The fourth-order Runge-Kutta equation is as follows:

6 $${\displaystyle \begin{array}{l}{\textrm{y}}_{\textrm{t}+1}={y}_{\textrm{t}}+\frac{h}{6}\left({k}_1+2{k}_2+2{k}_3+{k}_4\right),\\ {}{k}_1=f\left({x}_{\textrm{t}},{y}_{\textrm{t}}\right),\\ {}{k}_2=f\left({x}_{\textrm{t}}+\frac{h}{2},{y}_{\textrm{t}}+\frac{h}{2}{k}_{\textrm{t}}\right),\\ {}{k}_3=f\left({x}_{\textrm{t}}+\frac{h}{2},{y}_{\textrm{t}}+\frac{h}{2}{k}_2\right),\\ {}{k}_4=f\left({x}_{\textrm{t}}+\frac{h}{2},{y}_{\textrm{t}}+\frac{h}{2}{k}_3\right),\end{array}}$$

In Eq. 6, where *k*_*1*_*, k*_*2*_*, k*_*3*_, and *k*_*4*_ are the slopes of several points in the interval [x_t_, x_t + 1_], *k*_*1*_ is the slope at the beginning of the period, *k*_*2*_ and *k*_*3*_ are the slopes of the midpoint of the period, *k*_*4*_ is the slope of the end of the time, and *h* is the time interval. The next value, *y*_*t + 1*_, is determined by the product of the current value *y*_*t*_ plus the time interval h and the estimated slope. The *y* in the model can be calculated as *S*, *E*, *I*, and *R*, respectively. In the initial setup of the model, *S*_*0*_, *E*_*0*_, *I*_*0*_, and *R*_*0*_ represent the quantities of susceptible, latent, infected, and removed individuals, respectively. Here, *R*_*0*_ refers to the total number of individuals who no longer transmit the virus at the beginning of the model, which is different from the basic reproduction number used in epidemiology to describe the transmission capacity of infectious diseases. Based on the specified initial values, predicted values for the numbers of susceptible individuals (*S*_*t*_), latent individuals (*E*_*t*_), confirmed individuals (*I*_*t*_), and removed individuals (*R*_*t*_) can be obtained. Then the predicted value and the actual data of the epidemic are constructed. The sum of squares of residuals is shown in Eq. 7.

7 $$f= sum\left({\left({S}_{true}-S\right)}^2+{\left({E}_{true}-E\right)}^2+{\left({I}_{true}-I\right)}^2+{\left({R}_{true}-R\right)}^2\right),$$

In Eq. 7, *S*_*true*_*, E*_*true*_*, I*_*true*,_ and *R*_*true*_, respectively, represent the actual number of susceptible people, the number of latent people, the number of confirmed cases, and the number of transferred cases (including the number of cured cases and the number of dead cases), and *S, E, I* and *R* respectively represent the predicted number of susceptible people, the number of latent people, the number of confirmed cases and the number of transferred cases.

(3) The fmincon function in MATLAB was utilized to minimize the sum of squared residuals. For this purpose, suitable initial parameter values were established, and the feasible domain for these parameters was defined, ensuring that the values remained within a reasonable range throughout the estimation process. Through an iterative optimization algorithm, each step involved adjusting the parameter values based on the gradient information of the objective function at the current parameters, aiming to reduce discrepancies between the model output and actual epidemic data. The optimization process was continuous until the sum of squared residuals reached its minimum, at which point the parameter set formed the best fitting solution for the model, namely the fitted values of parameters *β*, *θ*, *α*, and *γ*. The constraints applied in the fmincon function are as follows:

8 $$\min f(x)\to {\displaystyle \begin{array}{l}\begin{array}{l}c(x)\le 0\\ {} ceq(x)=0\end{array}\\ {}A\cdot x\le b\\ {}\begin{array}{l} Aeq\cdot x= beq\\ {} lb\le x\le ub\end{array}\end{array}},$$

where *c(x)* is a nonlinear inequality, *ceq(x)* is a nonlinear equation, *A·x < =b* is a linear inequality, and *Aeq·x = beq* is a linear equation. Since there is no linear inequality constraint in the model, this paper sets *A = [], b = [], Aeq = [], Beq = []*, *lb,* and *ub* as the lower and upper bounds of the linear inequality constraint of variables. This paper sets parameters *β、θ、α* and *γ* range is [0,1].

Step 4: Build a relationship network. Constructing the social network of agents represents the interaction between agents and simulates the spread of viruses in cities.

Step 5: Acco*r*ding to the transmission mechanism of the agent, determine the infection rules of virus transmission.

Step 6: Use the experimental data to simulate and output the spatial distribution of the agent at the last moment after the simulation time and the curve of each agent over time.

### Design of the study

**Fig. 6:**
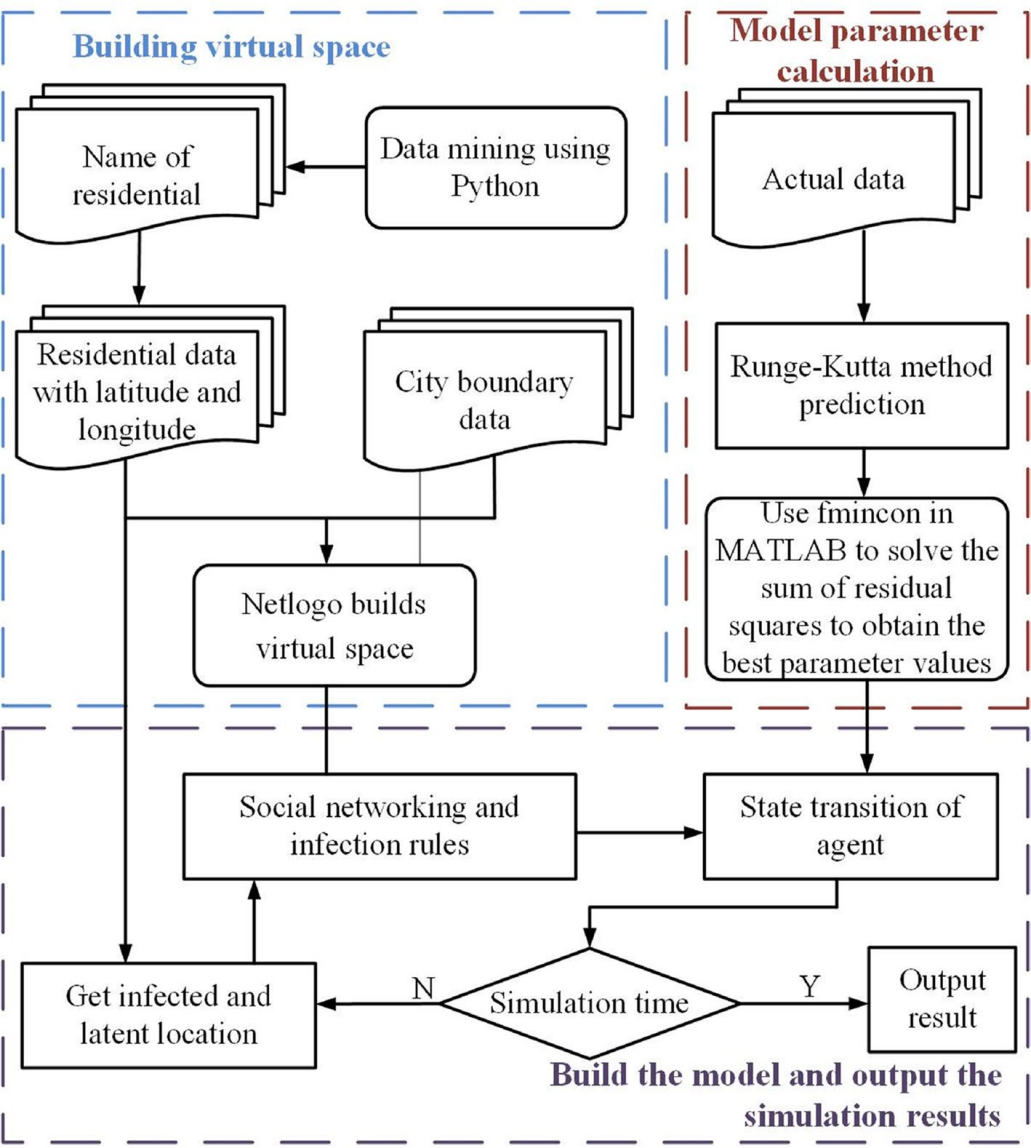
Multi-agent coordination propagation model of COVID-19 spatiotemporal spread

The main objective of this study is to simulate the spread of COVID-19 using a Multi-Agent Simulation approach integrated with the Small-World Network framework. The experimental design comprises several essential components to ensure the robustness and credibility of the research findings. Firstly, based on detailed neighborhood data in the main urban area of Wuhan, we established a virtual space containing latitude, longitude, and infection counts. This step involved using Python for data mining to obtain neighborhood names, locations, and COVID-19 infection case data. This data serves as the basis for building the Multi-Agent Simulation model. Secondly, model parameters are computed by utilizing the Runge-Kutta method to predict COVID-19 data, and the fmincon function in MATLAB is employed to obtain the optimal parameter values by minimizing the sum of squared residuals. Lastly, the Multi-Agent Simulation model is constructed, and simulation results are outputted. The position of infected and exposed individuals is determined from the previous steps, and social relationship networks and infection rules are established to simulate interactions among agents. By incorporating the transition rules, the state changes of agents over time are modeled, and simulations are conducted to output the results. And the implementation steps are further detailed in Fig. 6.

#### Study area and data processing

**Fig. 7:**
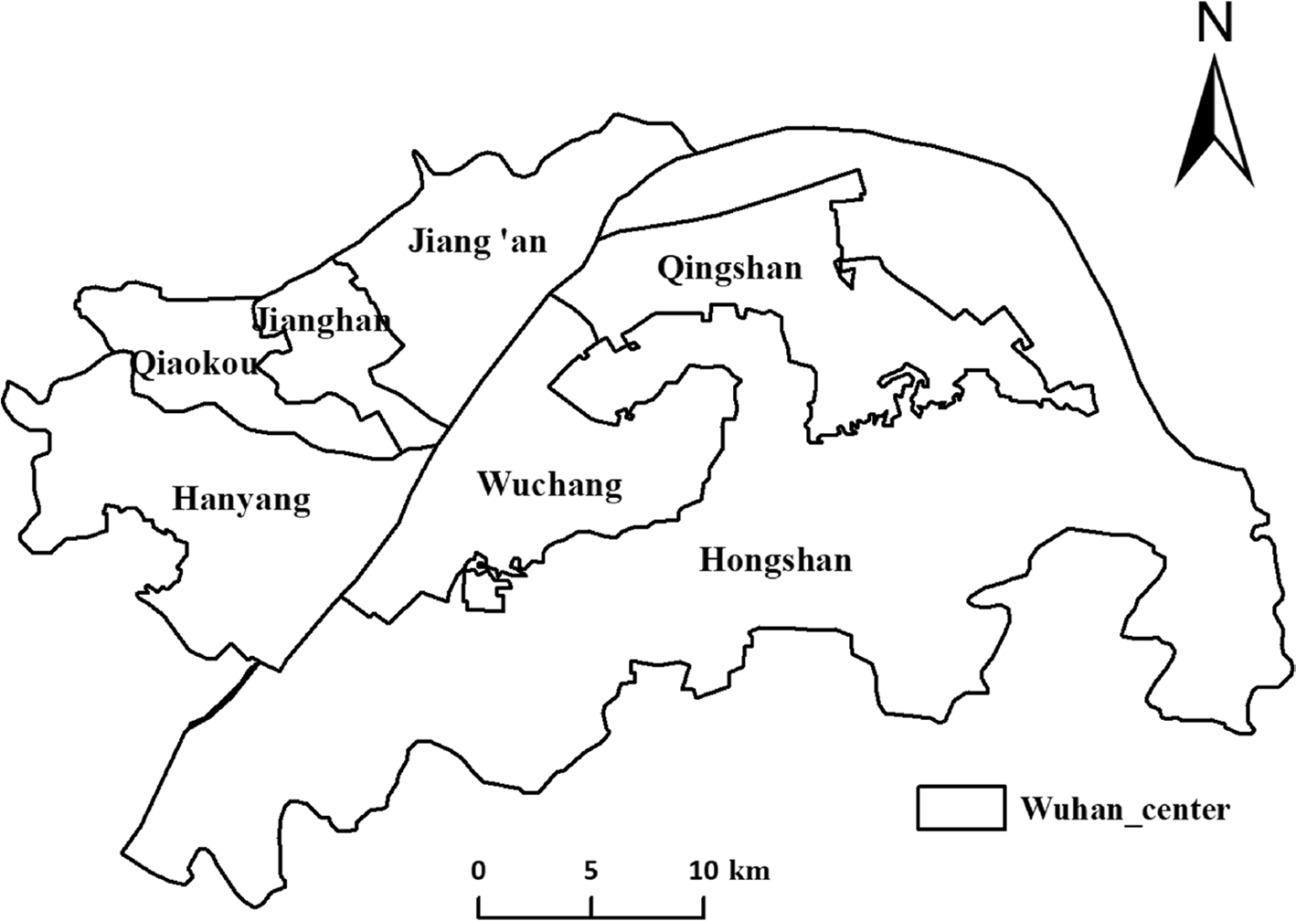
The central urban area of Wuhan

Wuhan was the city most seriously affected by the early stage of the COVID-19 epidemic in China. There are 13 districts in Wuhan, mainly including seven central urban areas of Jiang’an district, Jianghan district, Qiaokou district, Hanyang district, Wuchang district, Qingshan district, Hongshan district, and six administrative districts of Dongxihu district, Hannan district, Caidian district, Jiangxia district, Huangpi district, and Xinzhou district. Wuhan is China’s most significant inland water, land, and air transportation hub and the shipping center in the middle reaches of the Yangtze River. Its high-speed rail network radiates over half of China and is the only city in Central China that can directly travel to five continents worldwide. As of the end of 2020, Wuhan has an area of 8569.15 km^2^, a permanent population of 12.3265 million people, and a regional GDP of 1.56 trillion yuan. This paper selects the research area as the central urban area of Wuhan (as shown in Fig. 7).

**Fig. 8:**
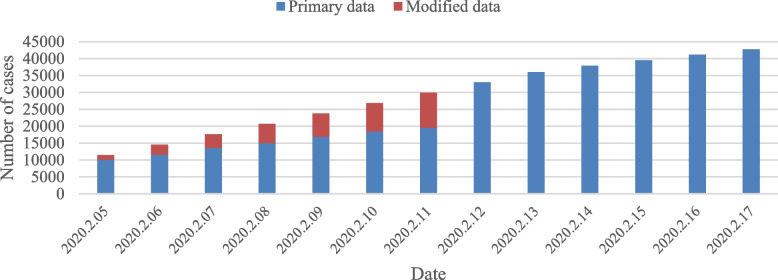
Comparison of raw and corrected data

The data used in the propagation model mainly include COVID-19 epidemic data and epidemic small-area data in the urban area of Wuhan. The COVID-19 epidemic data in Wuhan is sourced from the real-time data published around 7 PM on a daily basis by the DXY website (https://ncov.dxy.cn/), including daily confirmed cases, cumulative confirmed cases, recovered cases, and deaths. Due to the change in the diagnosis method for COVID-19 in Hubei Province on February 12th, about 12,000 clinical cases were added to the cumulative cases reported in Wuhan that day. In order to make the data more reasonable and reliable, the newly added data on February 12th was allocated to each day in the previous week according to the daily increase ratio of confirmed cases in the previous week [37], as shown in Fig. 8.

The epidemic small-area data in the urban area of Wuhan was obtained from the lists of Wuhan’s first and twentieth epidemic-free communities and villages, which were published by Changjiang Daily. Starting from March 6, 2020, the Neighborhood Prevention and Control Group of the Wuhan COVID-19 Prevention and Control Headquarters released 20 batches of lists containing areas, communities, and villages (teams) with no reported cases. The designation of a no-epidemic neighborhood is contingent upon meeting both the criteria of ‘zero cases’ and ‘comprehensive control’. Since the data from the twentieth publication showed that 99.9% of the communities and villages had no cases, we regarded the communities and villages from this publication as all of Wuhan’s residential areas. We obtained the location information of Wuhan’s residential areas by using the Amap API according to their names and converted the obtained latitude and longitude information into vector data. We then spatially connected the vector data of residential areas with the vector data of central urban areas of Wuhan. Finally, we obtained the residential area data for each district in the central urban area, as shown in Table 2. The epidemic residential areas were determined by comparing the first and twentieth epidemic-free neighborhood and village lists.

**Table 2:** Situation of epidemic residential areas in various districts of Wuhan

Area	Total number of plots	Epidemic plots	No epidemic plots	Proportion of epidemic plots
Jiang’an	1056	789	267	75%
Jianghan	668	491	177	74%
Qiaokou	489	365	124	75%
Hanyang	403	347	56	86%
Wuchang	848	424	424	50%
Qingshan	258	207	51	80%
Hongshan	949	857	92	90%
Sum	4671	3480	1191	75%

#### Parameter determination

When using epidemic models to study the spread of epidemics, the most critical issue is to determine the transmission parameters of the epidemic, including infection rate, transition rate, and removal rate. Since the outbreak of COVID-19, China has taken a series of measures to control the development of the epidemic, such as closing off communities, establishing a shelter hospital, and requiring temperature checks to enter public places. To ensure that the quantitative parameter values are closer to the actual values, we divided the epidemic into four stages based on three main time points during the Wuhan epidemic, namely the closure of traffic on January 23rd, the closure management of the neighborhood on February 10th, and the implementation of “bed waiting” on February 27th. The parameter fitting values for each stage are obtained according to Step 3, and the results are shown in Table 3.

**Table 3:** Quantitative values for each parameter at different stages

Time phasing	*β*	*θ*	*α*	*γ*
Stage I (2020.1.10–2020.1.22)	0.6101	0.1910	0.0803	0.0318
Stage II (2020.1.23–2020.2.09)	0.0001	0.3267	0.1843	0.0187
Stage III (2020.2.10–2020.2.26)	0.0001	0.1038	0.2803	0.0211
Stage IV (2020.2.27–2020.4.25)	0.0001	0.1282	0.3960	0.0761

**Fig. 9:**
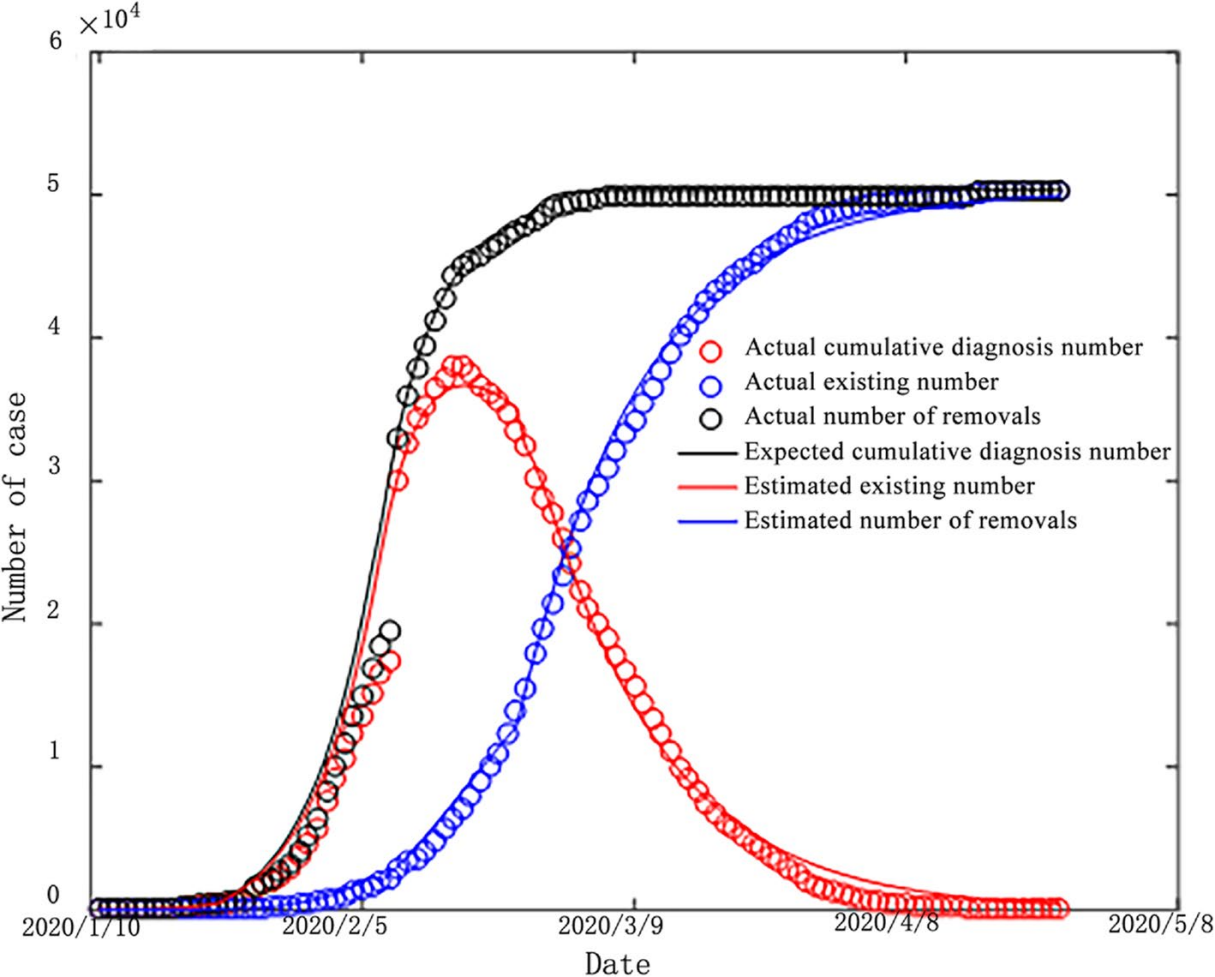
Comparison of SEIR model simulation results with actual values

After substituting the parameter values from Table 3 into the SEIR model, the fit curve obtained was compared with the actual epidemic data, as shown in Fig. 9. From the figure, it can be seen that the fitted values of cumulative confirmed cases, currently confirmed cases and removed cases show a similar trend to the actual epidemic data, indicating a good degree of model fitting and high accuracy of the quantified parameter fitting values in each stage.

Since the first place where the coronavirus was discovered and widely spread in Wuhan was the Huanan seafood market, we assumed it to be the initial infectious agent. Before January 23rd, 2020, the crowd was a regular activity, and the average network degree was 6. This setting is based on the theory of six degrees of separation, which posits that in social networks, any two individuals are, on average, connected by only five intermediaries (or six steps) to establish contact [38]. This theory has gained further support and development within the context of social networks and big data analysis [39, 40]. Additionally, the small-world network model effectively simulates real-world social networks’ structure, particularly in describing population clustering and social interactions [41, 42]; After January 23rd, Wuhan was “closed,” all public transportation was halted, and all long-distance connections were cut off. On February 10th, Wuhan carried out the closed management of the neighborhood, the social contact between the communities was cut off, and all network connections were removed. The outbreak began with the discovery of the first unexplained pneumonia case on December 8th, 2019, until the existing confirmed cases in Wuhan became zero on April 25th, 2020. The period lasted 140 days, and the model set the simulation time to 140.

## Results

**Fig. 10:**
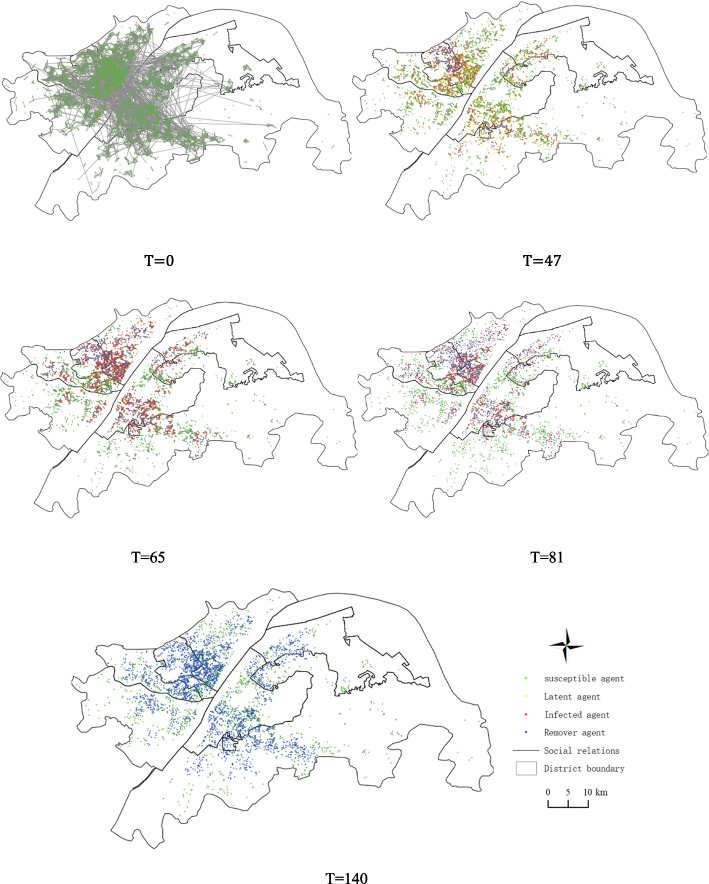
Spatial distribution of agents at each moment of the single simulation process

In the simulation process of the spatial and temporal spread of the COVID-19 epidemic, the spatial and temporal distribution of various agents in the first day and the end time of each stage of the four stages, namely T = 0, T = 47, T = 65, T = 81, T = 140, was recorded. Figure 10 shows the spatial distribution of agents at each moment in the single simulation process. T = 0 day is the initial moment of the simulation. Currently, there are only two types of agents: susceptible and infected. The susceptible agent is all the communities in the urban area of Wuhan, and the infected agent is the South China Seafood Market assumed in this paper. T = 47 days is January 23rd, Wuhan city closure day, infected agents infect susceptible agents through social networks. After a period of spread, most agents around the South China Seafood Market are infected through short connections into infected and latent agents. Some susceptible agents are removed after infection, and some long-distance agents are infected through long connections. The number of infected agents increased significantly at T = 65 days. Since Wuhan closed all public transport facilities on January 23rd, all long connections were removed. Most new infections were around the infected or latent agents at T = 47 days. T = 81 days, all the latent agents are transformed into the infected agent, because Wuhan on February 10th, all the neighborhood closed management, all connections were removed, and the epidemic did not further spread; at the end of the T = 140-day simulation, almost all the infected agents are converted to the emigrant agents.

**Fig. 11:**
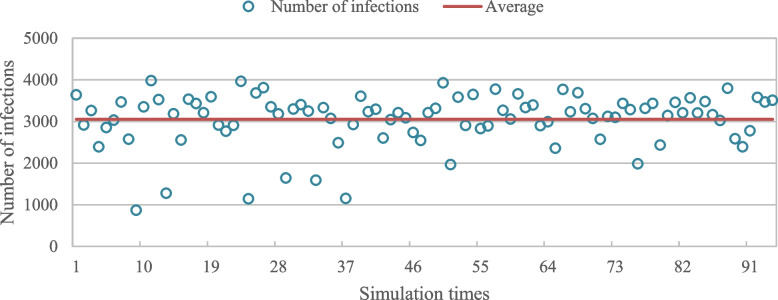
Simulation of 1 hundred cumulative outbreak plots

Because the spread of epidemic diseases is random in space and quantity, the results of a single simulation may not be representative. To address this, 1 hundred random simulations were conducted on the research scope using the same parameters, and the data where the epidemic did not successfully spread (initial patients were cured or died without spreading the virus to others) was removed. Ultimately, 94 sets of data were obtained. As depicted in Fig. 11, the simulation results show that the average number of infected agents is 3053, which differs by 12.27% from the actual number of infected communities in Wuhan (3480). After the simulation, the average number of infected agents is 10.

**Fig. 12:**
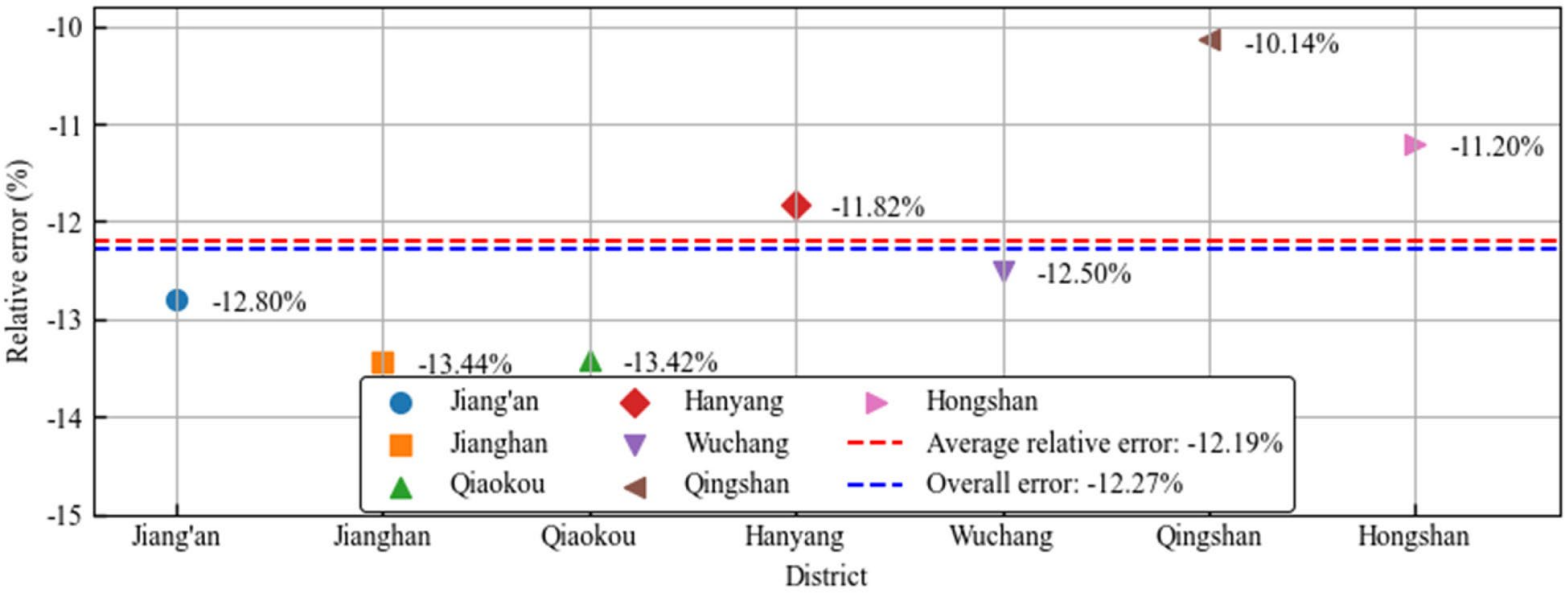
Regional error analysis chart

A detailed analysis of the data for various regions, as shown in Fig. 12, reveals the relative errors for different districts: Jiang’an District has a relative error of − 12.80%, Jianghan District is at − 13.44%, Qiaokou District at − 13.42%, Hankou District at − 11.82%, Wuchang District at − 12.50%, Qingshan District at − 10.14%, and Hongshan District at − 11.20%. Combining the data from these regions, the average relative error is found to be − 12.19%. This indicates that the results of this simulation closely align quantitatively with actual epidemiological data, thereby validating the effectiveness and reliability of the simulation method employed in predicting the dynamics of the COVID-19 virus transmission.

**Fig. 13:**
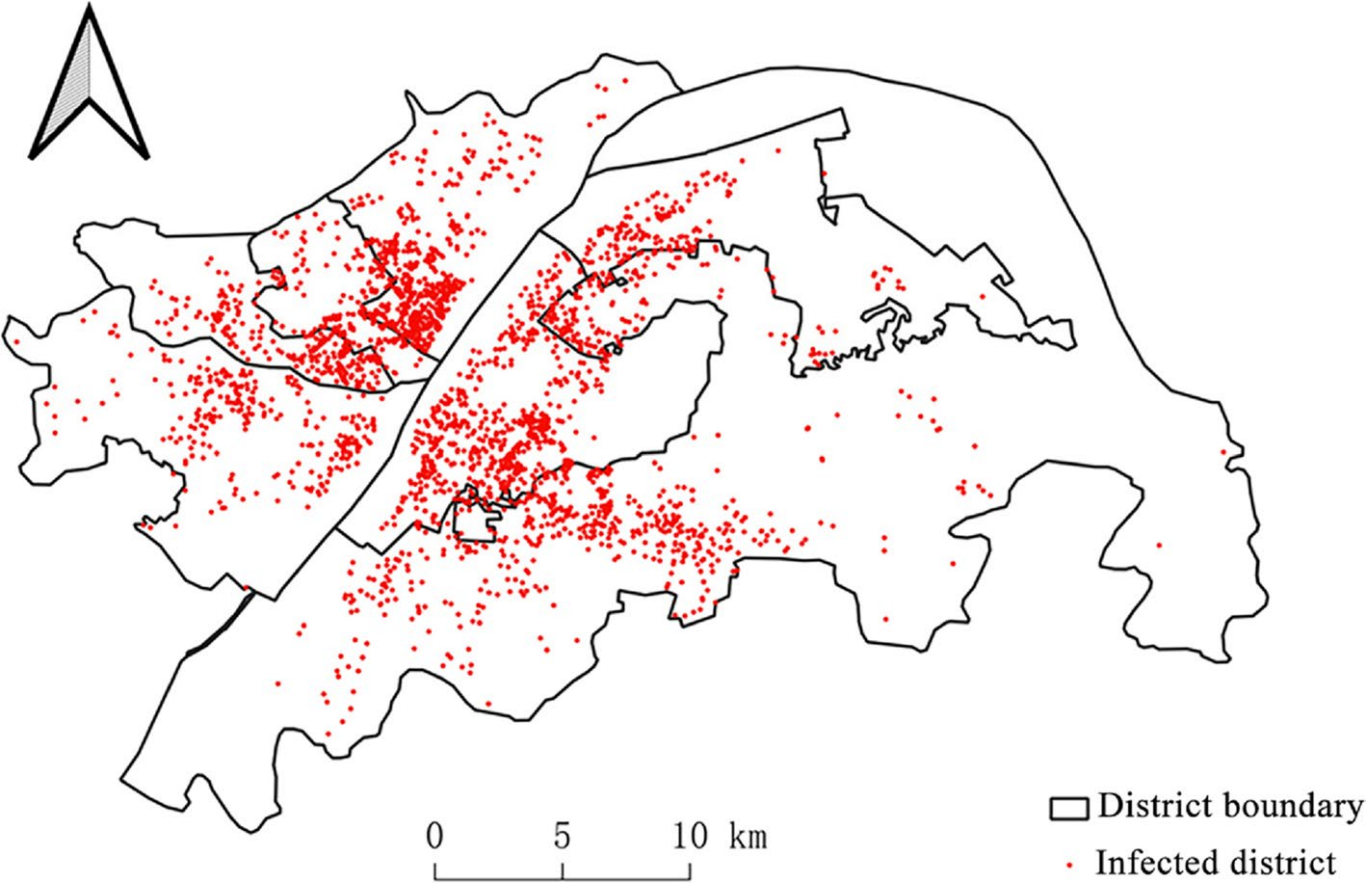
Distribution of outbreak plots in Wuhan

According to the results of the 20th non-epidemic area and the first non-epidemic area, the distribution map of the epidemic area (Fig. 13) was calculated. It can be seen from the map that the epidemic is concentrated in the urban center, where economic activities are frequent, and population flows are large. It can be found that there is a close relationship between COVID-19 and population density. The more opportunities for contact between people, the higher the prevalence of the epidemic.

**Fig. 14:**
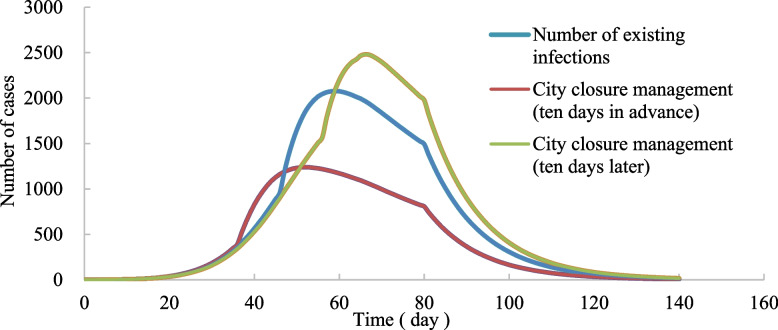
Epidemic trends under the three “city closure” time scenarios

In order to analyze the influence of the intervention effect of prevention and control measures on the epidemic development, 100 simulations were carried out for the scene of “closing the city” in Wuhan 10 days earlier and 10 days later, respectively. After each scenario simulation, the data were cleaned up, and finally, the valuable data were averaged as the epidemic spread result (Fig. 14). The simulation results show that the number of infected agents peaked at 2077 on the 59th day in the normal situation. However, the number of infected agents who “closed the city” 10 days in advance reached the maximum value of 1239 in 52 days, reaching the peak 7 days earlier than the typical scenario. The peak number of infected agents was 40.35% less than the typical scenario. However, infected agents peaked at 2480 on the 66th day, 7 days later than the standard scenario. The peak number of infected agents was 19.40% higher than that in the typical scenario. The intervention of prevention and control measures will significantly impact the spread of the epidemic. The earlier the intervention, the fewer the number of patients at the peak of the epidemic, and the effective control of the epidemic as soon as possible will have a more significant effect on restraining the spread of the epidemic.

## Discussion

The global COVID-19 pandemic has profoundly impacted socio-economic activities and public health. This study focuses on Wuhan with a daily time scale. We employed an integrated approach of intelligent agent-based modeling, complex network modeling, and GIS technology to build a COVID-19 spatial diffusion model. This model used the Runge-Kutta method with the SEIR model to fit critical parameters and was segmented into four stages based on early prevention measures in Wuhan. It successfully replicated COVID-19’s spatial diffusion in Wuhan’s main urban areas from December 8th, 2019, to April 25th, 2020, with experimental results closely matching actual observations.

This study used a multi-agent simulation technique based on small-world networks to simulate realistically the transmission characteristics of urban social networks. Small-world networks’ unique structure and connectivity capture social network complexity and simulate dynamic individual interactions, providing an accurate epidemic spread model. This model also flexibly demonstrates various control strategy effects, forming a theoretical basis for effective prevention and control measures.

Our data source is non-epidemic areas reported by the ‘Changjiang Daily,’ defined as regions without confirmed COVID-19 cases for at least 14 consecutive days, with the removal of suspected cases, fever cases, and close contacts. The first non-epidemic area data collection began on February 21st. Although there might be infections before March 6th, affecting infected areas and village counts in Wuhan districts, after a hundred simulations, the model closely approximates the actual value despite occasional abnormal predictions.

Simulation simplifies real-world behavior over time. However, the actual world is intricate and influenced by many variables. COVID-19’s spread is complex, and despite parameter quantification in stages, government prevention efforts and community and medical facility responses affect parameters like contact, infection, and removal rates. Regional differences in prevention and control measures make accurate model parameter quantification challenging. The stochastic model and averaging of 100 simulation results may differ from actual COVID-19 historical data, which is a random process.

This study significantly improved our biological understanding of COVID-19 spread by creating a spatial diffusion model for Wuhan. It reveals epidemic diffusion patterns under various prevention measures, demonstrating virus transmission mechanisms in diverse social and environmental conditions. Predicted results provide a scientific basis for evaluating and enhancing public health responses, guiding future epidemic control. Despite effectively simulating COVID-19 spread in Wuhan, the model has limitations, primarily relying on fixed social networks and neglecting complex population mobility and social interactions. Additionally, it does not cover other potential preventive measures like isolation and medical resource allocation, suggesting a need for future research to enhance model comprehensiveness and practicality.

## Conclusions

Since viruses mostly spread through fixed social networks, simulating the pandemic using micro-individuals within urban spatial structures has limited significance. Thus, the neighborhood is treated as an agent with traits and behavioral rules, and a cyberspace with small-world characteristics represents the social connections among agents. The proposed model of COVID-19 spatiotemporal spread of small-world network collaborative multi-agent explicitly considers the influence of the distance between agents and social relations on epidemic spread. Using the NetLogo platform, the spatiotemporal spread process of COVID-19 is simulated in the Wuhan area. The difference between the simulation and actual results is 12.27%, which shows that the model can effectively illustrate the spread law of COVID-19 in urban space.

The propagation model also examines the epidemic changes under various scenarios, revealing that prevention and control measures can significantly inhibit epidemic spread. Earlier implementation of these measures leads to a more pronounced effect on inhibiting the disease’s transfer. The outcomes of different scenarios tested by the model may help enhance the safety prevention and control system of urban tectonic space.

## Data Availability

The data that support the findings of this study are available from the corresponding author upon reasonable request.
